# Heterotopic Ossification After COVID-19: A Case Report and Review of Literature

**DOI:** 10.7759/cureus.84539

**Published:** 2025-05-21

**Authors:** Hidetoshi Tsugeno, Tetsuro Hida, Yoshitoshi Higuchi, Toshihiro Ando, Koji Sato

**Affiliations:** 1 Department of Orthopaedic Surgery, Japanese Red Cross Aichi Medical Center Nagoya Daini Hospital, Nagoya, JPN

**Keywords:** covid-19, heterotopic ossification, myositis ossificans, pregnancy, sars-cov-2

## Abstract

We report a 31-year-old woman hospitalized for approximately two months for coronavirus disease 2019 (COVID-19) pneumonia. Under sedation, she received physiotherapy to prevent joint contractures. When weaning from the mechanical ventilator began, she complained of pain and limited range of motion in her bilateral hip and knee joints. Radiography and computed tomography revealed non-traumatic heterotopic ossification (HO) in the bilateral vastus medialis and hips. Range-of-motion exercises were discontinued, and treatment with indomethacin and etidronate disodium was started. Alkaline phosphatase, an index of disease activity, peaked 15-fold higher than the normal range but decreased to near-normal levels four months after treatment. She could walk with a T-cane after rehabilitation, although her range of motion remained limited. If a COVID-19 patient has joint pain and is immobilized for a long time, HO should be considered accordingly.

## Introduction

Heterotopic ossification (HO) is a phenomenon in which bone forms abnormally in areas where bone tissue does not normally exist, such as muscles, fascia, ligaments, and joint capsules. The most common sites for HO to occur are the pelvis, hip, knee, shoulder, and elbow joints. It is often caused by musculoskeletal trauma, surgery, burns, nervous system injury, immobilization, and inborn and metabolic diseases [1]. Recently, two case series have been published demonstrating HO around the shoulder and hips of coronavirus disease 2019 (COVID-19) patients who required mechanical ventilation [2,3]. We present the case of a patient who developed HO around multiple joints after a prolonged hospitalization for COVID-19.

## Case presentation

**Figure 1:**
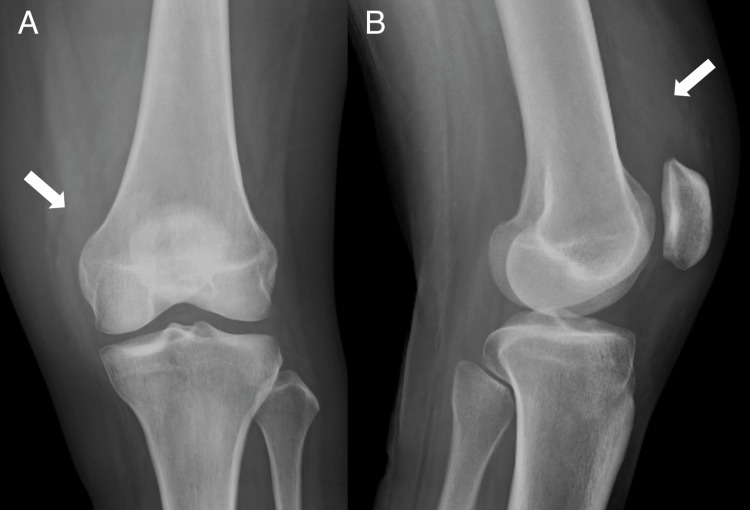
Left knee anteroposterior (A) and lateral (B) X-ray. Immature heterotopic ossification (HO) is identified in the medial knee (arrows). Immature heterotopic ossification in the medial knee (arrows).

**Figure 2:**
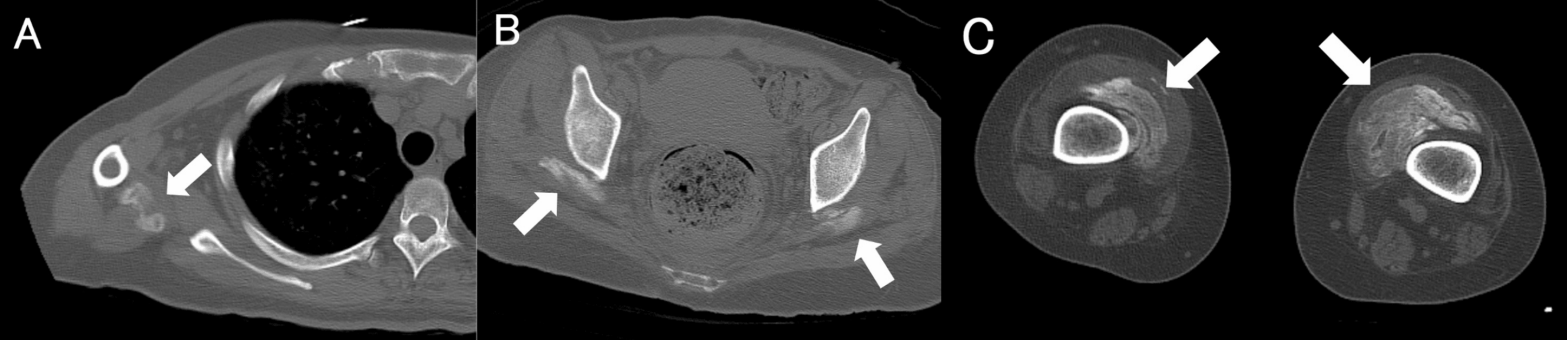
Whole-body computed tomography of immature heterotopic ossification (HO) in the right triceps brachii (A), bilateral gluteus medius (B), and vastus medialis (C).

**Figure 3:**
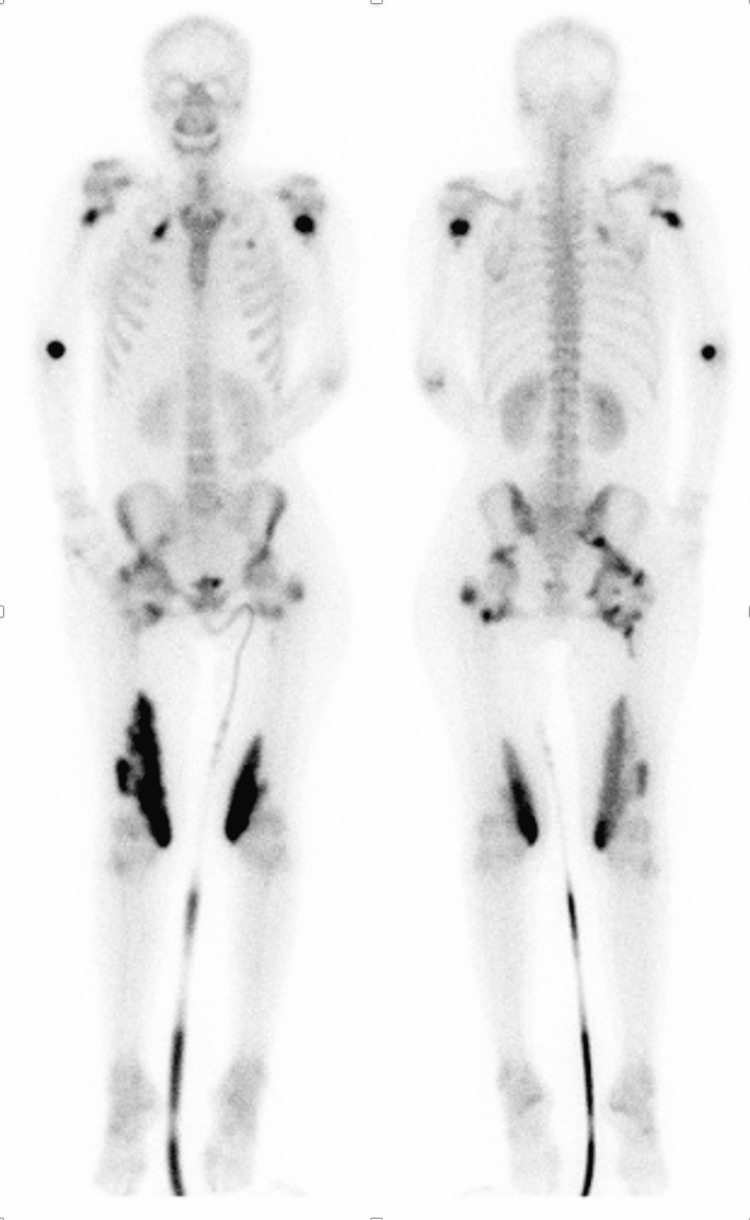
Bone scintigraphy showing hyperfixation of the bilateral axillae, hip, vastus medialis, and right elbow.

We report a case of a 31-year-old pregnant woman. She developed gestational diabetes mellitus during pregnancy, and insulin therapy was initiated. She was admitted to the hospital for COVID-19 at 33 weeks of gestation. On day 2, the patient's respiratory status worsened, and a cesarean section was performed. On day 3, favipiravir and methylprednisolone were administered. On day 5, the patient was intubated in the intensive care unit as a result of her worsening respiratory status and was subsequently treated with remdesivir until day 13. The patient's respiratory status, however, did not improve, and this was accompanied by worsening inflammation. We then considered the possibility of ventilator-associated pneumonia and started piperacillin/tazobactam administration from day 7. The patient’s condition continued to deteriorate and her treatment was therefore managed with extracorporeal membrane oxygenation (ECMO) from day 10. We continued to monitor the patient's progress with broad-spectrum antibiotics, but her improvement was poor. One month after admission, *Candida orthopsilosis* was detected in her blood culture and she was treated for candidemia. Simultaneously, the patient's thrombocytopenia progressed and she required a daily platelet transfusion. The cause of the low platelets was proposed to be disseminated intravascular coagulation associated with candidiasis and heparin-induced thrombocytopenia (HIT) as her HIT antibody test was weakly positive. In parallel with the treatment of the fungal infection, the heparin, which was used as an anticoagulant, was changed to argatroban. Even after the introduction of ECMO, the lung lesions did not significantly improve, and a living donor lung transplant was considered. This treatment plan was abandoned due to a lack of compatibility with the donor. Nevertheless, two months after admission, her lung field lesions improved rapidly, and the patient was weaned off ECMO. When weaning of sedation began, the patient started to experience pain in her hips and resistance during joint mobilization exercises. Because of the prominent limitation in her range of motion (ROM) in both hip and knee joints, radiography, whole-body computed tomography (CT), and bone scintigraphy were performed. This revealed evidence of ossification around both shoulder joints, the right anterior chest, both hips, and both knees. A diagnosis of non-traumatic HO was made (Figures 1-3).

**Figure 4:**
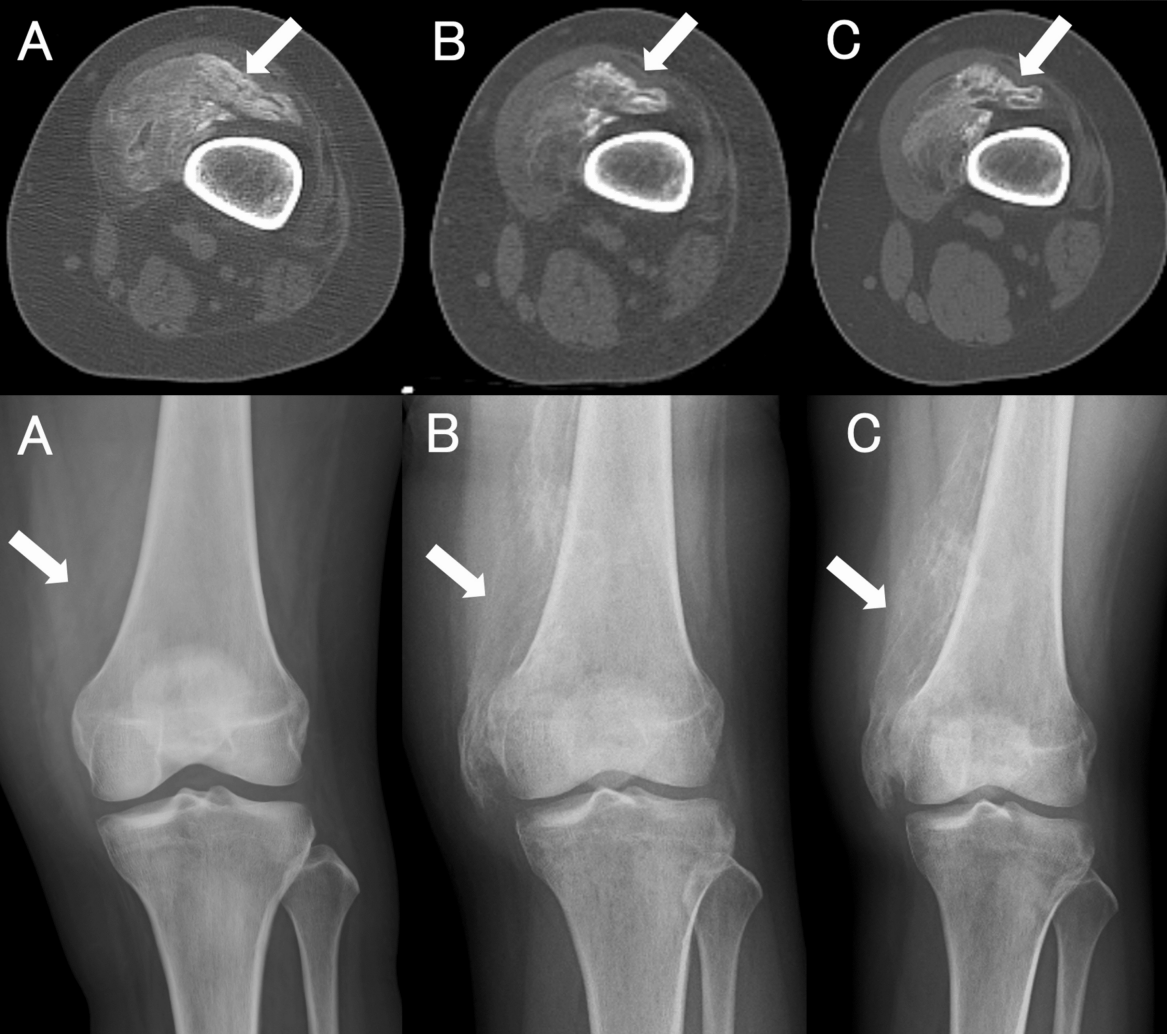
Changes over time in the left femur computed tomography scan (top panel) and X-ray image (bottom panel). A: Onset, B: Two months later, C: Four months later. The soft-tissue ossification shows well-developed cortex and medullary spaces (arrows).

Active ROM exercises were discontinued, and the patient was treated with indomethacin and etidronate disodium. Alkaline phosphatase (ALP) levels can be used to assess HO. During hospitalization, her highest level of serum ALP was 1976 IU/L (normal range: 38-113 IU/L). Her serum calcium levels had a minimum value of 7.3 mg/dL. Her serum phosphorus levels fluctuated and exceeded both the upper and lower limits of the normal range. Her renal function remained normal throughout the disease. Four months after admission, the patient was transferred to the hospital for rehabilitation. By that time, she was able to walk with the assistance of a walker. Two months after discharge from our hospital, she was discharged from the rehabilitation hospital and returned to her home. At that time, her HO had slightly improved on a CT scan, but four months after her discharge from the hospital, her HO had worsened (Figure 4).

She was, however, able to walk with a T-cane. In addition, her ALP levels improved and dropped to 201 IU/L. A comparison between previous studies and this study is presented in Table 1.

**Table 1:** Clinical characteristics of heterotopic ossification in patients with COVID-19 that have been documented thus far HT: hypertension; COPD: chronic obstructive pulmonary disease; ALP: alkaline phosphatase (normal range: 38–113 IU/L); DM: diabetes mellitus; WNL: within normal limit

Reference	Age (years)	Sex	Comorbidities	Duration of mechanical ventilation	Location	Days to diagnosis	ALP (IU/L)
Meyer et al. [2]	64	M	HT, atrial fibrillation, cervical myelopathy	26 days	Bilateral hip	39 days	200
Meyer et al. [2]	73	M	HT, COPD	27 days	Left hip	40 days	126
Meyer et al. [2]	74	M	HT, COPD	30 days	Left hip	41 days	105
Meyer et al. [2]	39	M	Schizophrenia, bipolar disorder, alcohol abuse	28 days	Bilateral shoulder	30 days	200
Aziz et al. [3]	51	F	HT, DM	Approx. 78 days	Bilateral shoulder	5.5 months	148
Aziz et al. [3]	43	F	HT	Unclear	Bilateral shoulder	6 months	WNL
Current case	31	F	pregnancy	83 days	Polyarticular	74 days	1976

## Discussion

HO, in which bone forms outside the skeletal system, is usually associated with paralysis and immobilization after trauma, nervous system injury, acute respiratory distress syndrome (ARDS), surgery, or burns [4,5]. The etiology is still unclear and is thought to be caused by neuromuscular blockade, electrolyte abnormalities, disruption of calcium homeostasis, microhemorrhage, osteoporosis, and muscle atrophy [5,6]. The main complications of HO are a limited ROM in the involved area and peripheral neuropathy [1,7]. In a previous report, HO was observed in 5% of patients with ARDS who received intensive care [8,9]. In the present case, we believe that prolonged immobilization resulting from prolonged sedation and neuromuscular blockade from severe ARDS caused the development of HO. However, other factors such as abnormal calcium metabolism caused by the SARS-CoV-2 virus, prolonged systemic inflammation, and microinjury from joint mobilization exercises under sedation may have also contributed.

Burn injury and the resulting severe hypoxic environment promote the production of hypoxia-inducible factor 1α, which leads to angiogenesis as a result of increased levels of vascular endothelial growth factor. Angiogenesis is an important step in bone formation [10]. In this case, the patient also suffered from local tissue hypoxia, which may have contributed to the systemic development of HO.

During pregnancy, ALP is known to gradually increase, reaching a peak in the second trimester, about twice its pre-pregnancy value. Placental ALP isoenzymes account for most of this increase. This makes it difficult to differentiate between bone and biliary system diseases during pregnancy. However, it generally peaks with childbirth and declines thereafter [11,12]. In this case, the peak is around two months postpartum (the onset of HO), so it is unlikely to be placental-derived ALP, thus the ALP reflects the disease status of HO.

The treatment of HO is difficult. Once the diagnosis of early HO is confirmed, passive ROM exercises to maintain joint mobility are recommended. More active joint manipulation has been suggested, although the trauma resulting from this approach carries the risk of aggravating the condition [7]. Nonsteroidal anti-inflammatory drugs have been suggested as a prophylactic treatment, but there are advantages and disadvantages to using bisphosphonates [10]. Early and continuous mobilization is essential, and surgical resection is recommended when joint restrictions affect autonomy and quality of life. Surgical resection should only be performed in advanced cases of ossification [1]. Early rehabilitation is essential for the management of HO and COVID-19.

## Conclusions

Based on the results of our study, HO should be considered as a potential complication in COVID-19 patients with joint pain and immobilization. Early diagnosis and management of this condition are essential to minimize functional impairment. However, our study had several limitations, including a small study population, specific conditions of the study, and the purely observational nature of the study. Therefore, further larger, randomized studies are necessary to confirm our findings and expand our knowledge on this topic. Future research should also investigate effective prevention and management strategies for HO in COVID-19 patients.
